# First case report of myopericarditis linked to *Campylobacter coli* enterocolitis

**DOI:** 10.1186/s12879-016-2115-9

**Published:** 2017-01-05

**Authors:** Cameron R. M. Moffatt, Soniah B. Moloi, Karina J. Kennedy

**Affiliations:** 1 National Centre for Epidemiology and Population Health, Research School of Population Health, Australian National University, Canberra, 2602 ACT Australia; 2 Department of Cardiology, Canberra Hospital and Health Services, Canberra, 2605 ACT Australia; 3 Department of Microbiology, Canberra Hospital and Health Services, Canberra, 2605 ACT Australia

**Keywords:** *Campylobacter infections*, *Campylobacter coli*, *Myopericarditis*, *Etiology*, *Female*, *Case report*

## Abstract

**Background:**

*Campylobacter* spp. are a common cause of mostly self-limiting enterocolitis. Although rare, pericarditis and myopericarditis have been increasingly documented as complications following campylobacteriosis. Such cases have occurred predominantly in younger males, and involved a single causative species, namely *Campylobacter jejuni*. We report the first case of myopericarditis following *Campylobacter coli* enterocolitis*,* with illness occurring in an immunocompetent middle-aged female.

**Case presentation:**

A 51-yo female was admitted to a cardiology unit with a 3-days history of chest pain. The woman had no significant medical history or risk factors for cardiac disease, nor did she report any recent overseas travel. Four days prior to the commencement of chest pain the woman had reported onset of an acute gastrointestinal illness, passing 3–4 loose stools daily, a situation that persisted at the time of presentation. Physical examination showed the woman’s vital signs to be essentially stable, although she was noted to be mildly tachycardic. Laboratory testing showed mildly elevated C-reactive protein and a raised troponin I in the absence of elevation of the serum creatinine kinase. Electrocardiography (ECG) demonstrated concave ST segment elevations, and PR elevation in aVR and depression in lead II. Transthoracic echocardiogram (TTE) revealed normal biventricular size and function with no significant valvular abnormalities. There were no left ventricular regional wall motion abnormalities. No pericardial effusion was present but the pericardium appeared echodense. A diagnosis of myopericarditis was made on the basis of chest pain, typical ECG changes and troponin rise. The chest pain resolved and she was discharged from hospital after 2-days of observation, but with ongoing diarrhoea. Following discharge, a faecal sample taken during the admission, cultured *Campylobacter* spp. Matrix assisted laser desorption ionization time-of-flight (Bruker) confirmed the cultured isolate as *C. coli*.

**Conclusion:**

We report the first case of myopericarditis with a suggested link to an antecedent *Campylobacter coli* enterocolitis. Although rare, myopericarditis is becoming increasingly regarded as a complication following campylobacteriosis. Our report highlights potential for pericardial disease beyond that attributed to *Campylobacter jejuni*. However uncertainty regarding pathogenesis, coupled with a paucity of population level data continues to restrict conclusions regarding the strength of this apparent association.

## Background

*Campylobacter* species are among the most widespread and commonest cause of bacterial enterocolitis worldwide [1]. *C. jejuni* is recognised as the leading cause of human illness, although *C. coli* might account for up to 25% of *Campylobacter* enterocolitis [2]. Complications such as bacteraemia or the development of post-infectious sequelae have an established place in the epidemiology of campylobacteriosis, with cardiac complications being less commonly reported [1]. We describe what we believe to be the first case of myopericarditis associated with *C. coli* enterocolitis and discuss the epidemiology and pathogenesis of this condition.

## Case presentation

A 51-yo female was admitted to the cardiology unit of an Australian public hospital with a 3-day history of continuous, dull, non-radiating, non-pleuritic, left lateral chest pain, with associated diaphoresis, nausea, palpitations and pre-syncope. Some relief of chest pain was achieved by leaning forward. Administration of combined paracetamol and codeine phosphate also relieved the pain. Four days prior to the commencement of chest pain the woman had reported onset of an acute gastrointestinal illness, passing 3–4 loose stools daily. This diarrhoea was persistent at the time of presentation. The woman had no significant medical history or risk factors for cardiac disease, nor did she report any recent overseas travel.

Physical examination showed the woman to be afebrile but mildly tachycardic, with a heart rate of 106 bpm. Blood pressure was 120/76 mm Hg. Examination of the chest revealed normal heart sounds, without any murmur or rub. Jugular venous pressure was normal. Pulmonary examination was normal with no signs of respiratory distress (RR 18), although mild tachypnea was noted during the course of her admission. The abdomen was soft and non-tender.

Laboratory testing showed a mildly elevated C-reactive protein (30.0 mg/L; normal range <5.0 mg/L) and a raised troponin I (maximal value 70 ng/L at the time of emergency department presentation; normal range <16) in the absence of elevation of the serum creatinine kinase. Liver enzymes were mildly elevated with an ALT 65 U/L (normal range <55 U/L) and ALKP 142 U/L (normal range 20–110 U/L). Full blood count and serum electrolytes were unremarkable.

**Fig. 1:**
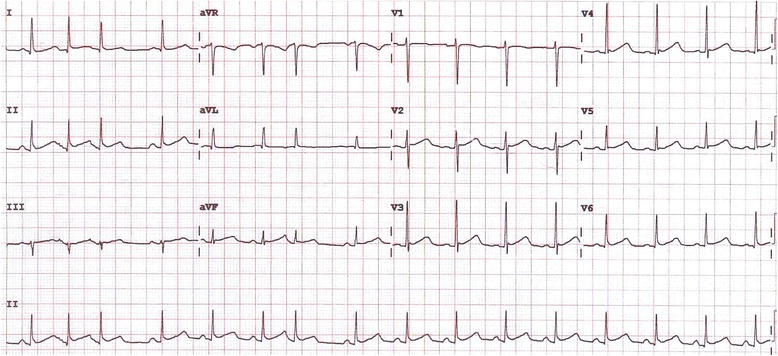
Electrocardiogram of case patient showing ST segment elevations in leads I, II, III, aVF and V6 and PR segment depression in lead II

Serial electrocardiographs demonstrated concave ST segment elevations involving leads I, II, III, aVF and V6, PR elevation in aVR and PR depression in lead II (Fig. 1). Differential diagnoses of completed acute myocardial infarction or pericarditis secondary to recent diarrhoeal illness were made, with the woman initially managed for an acute coronary syndrome with subcutaneous clexane 70 mg bd, aspirin 100 mg daily, clopidogrel 75 mg daily and metoprolol 25 mg bd prior to expert cardiology review and echocardiogram. Transthoracic echocardiogram (TTE) revealed normal biventricular size and function with no significant valvular abnormalities. No pericardial effusion was present but the pericardium appeared echodense and thickened. A diagnosis of myopericarditis was made.

Clexane and clopidogrel were ceased and colchicine 500mcg bd commenced. The chest pain resolved and she was discharged from hospital after 2 days, but with ongoing diarrhoea. Following hospital discharge, a faecal sample taken during the admission, cultured *Campylobacter* spp. in isolation. Matrix assisted laser desorption ionization time-of-flight (Bruker) confirmed the cultured isolate as *C. coli*.

## Discussion

Acute pericarditis is a not uncommon, frequently idiopathic disorder that mostly follows a benign clinical course. Some myocardial involvement often accompanies acute pericarditis, with the term myopericarditis indicating the pericardium as the primary focus [3]. Viruses are often implicated when acute pericarditis or myopericarditis are linked to infection, with bacterial causes less commonly described [3]. Diagnostic criteria for acute pericarditis include chest pain, pericardial friction rub, ST segment elevations and pericardial effusion, with a clinical diagnosis made when two of these criteria are met [3]. A clinical diagnosis of myopericarditis includes the additional detection of elevated cardiac enzymes or a new onset of depressed left ventricular systolic function [3].

*Campylobacter* spp. have an uncommon but increasingly recognised link with myopericarditis, as supported by a growing body of case reports and reviews of literature [4–6]. Our report is notable in terms of the observed differences in both pathogen and host characteristics. Firstly, our patient was positive for *C. coli*, a species well recognised as a cause of enterocolitis, but not previously reported in association with myopericarditis. Secondly, the patient was a healthy, middle-aged female, in contrast to previous patients where most were younger immunocompetent males [4–6]. Although *Campylobacter* does show tendency to cause higher rates of disease in men compared to women, the apparent predilection towards men in myopericarditis is striking. However despite this observation, epidemiological evidence of a causal association between campylobacteriosis and myopericarditis is lacking. We identified a single study that showed a greater incidence of myocarditis in *Campylobacter* positive cases than controls at 16.1 cases per 100,000 person-years (95% CI 2.3–114.4) compared to 1.6 cases per 100,000 person-years (95% CI 0.2–11.4), but the result was not significant and absolute case numbers were small [7].

It is important to make distinction between myopericarditis associated with *C. jejuni* or *C. coli* and that with *C. fetus*. The former are recognised causes of enteric disease in humans and likely represent archetypal causes of *Campylobacter* myopericarditis. Conversely *C. fetus* is an atypical, invasive species, capable of evading the host immune system by means of complement resistant surface proteins [8]. As such it is more frequently isolated from blood and pericardial fluid, with enteric symptoms either absent or less pronounced [9]. For *C. jejuni* (and *C. coli*) associated myopericarditis, the putative mechanisms include direct infection of the pericardium or myocardium, an immune hypersensitivity reaction, or the effect of bacterial toxins [10]. Currently evidence for direct infection is limited to two reports of *C. jejuni* detection in pericardial fluid [11, 12]. A dominant immune-mediated response also seems less likely given the short window between the onset of enteric and cardiac symptoms, as distinct to that seen with *C. jejuni* and development of other immune-mediated disease (e.g., Guillain–Barré syndrome and reactive arthritis) [10]. Although there is a lack of evidence for a specific *Campylobacter*-associated cardiotoxin [13], the short window period surely favors a toxin-mediated mechanism [4, 6]. Furthermore, the infrequency of *C. coli* myopericarditis as highlighted by our case might possibly be explained by the dominance of *C. jejuni* at the species level, along with the lower incidence of cytotoxin and enterotoxin production by *C. coli* compared to *C. jejuni* [13].

It is unclear whether antibiotic treatment influences the outcome of *Campylobacter* spp. myopericarditis, however a recent examination of *C. jejuni*-associated myopericarditis cases showed treatment with either macrolide or fluoroquinolone antibiotics to be a common practice although without any apparent consensus on dosage and treatment duration [5]. However campylobacteriosis is generally regarded as a self-limiting illness with antimicrobial therapy recommended for immunocompromised patients, patients whose symptoms are severe or persistent, or who develop extra-intestinal infections [1]. For our case confirmation of *Campylobacter* spp. in stool occurred following hospital discharge, with a mechanism for myopericarditis such as direct infection or toxin-mediated response not able to be readily determined. As such antimicrobial therapy was not commenced. Treatment for the myopericarditis was conservative, using nonsteroidal anti-inflammatory drugs (NSAIDS) and colchicine. Among case reports describing *Campylobacter*-associated myopericarditis we identified a single episode where colchicine, as an adjunctive to aspirin, was used in treating myopericarditis, with the case reported as fully recovered at a 3-month review [14]. Anecdotally, clinical outcomes for *C. jejuni* (and *C. coli*) associated myopericarditis appear favourable [5] with low rates of morbidity, mortality, evolution to heart failure and worsening ventricular function [15].

## Conclusion

We report the first case of myopericarditis linked to a *C. coli* enterocolitis, with the illness running a benign clinical course. While increasingly recognised as a complication of campylobacteriosis, the paucity of population level data restricts further conclusions on the strength of any association between *Campylobacter* infection and myopericarditis. Further, the mechanism(s) of pathogenesis also remain uncertain, although anecdotally may favour a toxin-mediated response.

## Abbreviations

ALKP: Alkaline phosphatase
ALT: Alanine transaminase
CI: Confidence interval
ECG: Electrocardiography
RR: Respiratory rate
TTE: Trans-thoracic echocardiography

## References

[1] Kaakoush NO, Castano-Rodriguez N, Mitchell HM, Man SM (2015). Global epidemiology of campylobacter infection. Clin Microbiol Rev.

[2] Man SM (2011). The clinical importance of emerging campylobacter species. Nat Rev Gastroenterol Hepatol.

[3] Imazio M, Trinchero R (2008). Myopericarditis: etiology, management, and prognosis. Int J Cardiol.

[4] Hannu T, Mattila L, Rautelin H, Siitonen A, Leirisalo-Repo M (2005). Three cases of cardiac complications associated with campylobacter jejuni infection and review of the literature. Eur J Clin Microbiol Infect Dis.

[5] Hessulf F, Ljungberg J, Johansson PA, Lindgren M, Engdahl J (2016). Campylobacter jejuni-associated perimyocarditis: two case reports and review of the literature. BMC Infect Dis.

[6] Kotilainen P, Lehtopolku M, Hakanen AJ (2006). Myopericarditis in a patient with campylobacter enteritis: a case report and literature review. Scand J Infect Dis.

[7] Becker S, Ejlertsen T, Kristensen B, Norgaard M, Nielsen H (2007). Is the incidence of perimyocarditis increased following campylobacter jejuni infection?. Eur J Clin Microbiol Infect Dis.

[8] Alzand BS, Ilhan M, Heesen WF, Meeder JG (2010). Campylobacter jejuni: enterocolitis and myopericarditis. Int J Cardiol.

[9] Morrison VA, Lloyd BK, Chia JK, Tuazon CU (1990). Cardiovascular and bacteremic manifestations of campylobacter fetus infection: case report and review. Rev Infect Dis.

[10] Uzoigwe C (2005). Campylobacter infections of the pericardium and myocardium. Clin Microbiol Infect.

[11] Fradejas I, Lopez-Medrano F, González-Montes E, Orellana A, Chaves F (2015). Campylobacter jejuni pericarditis in a renal transplant recipient on sirolimus therapy. Clin Microbiol Newsl.

[12] Rafi A, Matz J (2002). An unusual case of campylobacter jejuni pericarditis in a patient with X-linked agammaglobulinemia. Ann Allergy Asthma Immunol.

[13] Wassenaar TM (1997). Toxin production by campylobacter spp. Clin Microbiol Rev.

[14] Hull SR, Varma MP (2011). Myopericarditis following campylobacter infection. Ir J Med Sci.

[15] Imazio M, Brucato A, Spodick DH, Adler Y (2014). Prognosis of myopericarditis as determined from previously published reports. J Cardiovasc Med (Hagerstown).

